# Impact of COVID-19 epidemic on temporal pattern of new HIV diagnoses in Italy, 2021 database

**DOI:** 10.1093/eurpub/ckad156

**Published:** 2023-08-31

**Authors:** Maria Dorrucci, Vincenza Regine, Lucia Pugliese, Barbara Suligoi

**Affiliations:** Department of Infectious Diseases, Istituto Superiore di Sanità, Rome, Italy; Department of Infectious Diseases, Istituto Superiore di Sanità, Rome, Italy; Department of Infectious Diseases, Istituto Superiore di Sanità, Rome, Italy; Department of Infectious Diseases, Istituto Superiore di Sanità, Rome, Italy

## Abstract

**Background:**

New HIV diagnoses in Italy decreased drastically in 2020 due to COVID-19 related effects: 50% fewer diagnoses were reported by the National HIV Surveillance System. COVID-19 pandemic impact on HIV surveillance is unclear. We estimated the expected number of new HIV diagnoses in 2020 in order to isolate the impact of the COVID-19 pandemic.

**Methods:**

We analyzed 29 697 new HIV infections diagnosed from 2012 to 2020, reported to the National HIV Surveillance System. We assessed temporal trends of new HIV diagnoses applying negative binomial mixed effects models. We estimated the COVID-19 impact as the difference between the model-estimated slopes from 2012 to 2019 and the change reported in the diagnoses. The expected number of new HIV diagnoses in 2020 was also estimated and compared with the reported count.

**Results:**

Based on the historical trend, we expected a 15% (95% CI: 5–25%) decline of new HIV diagnoses in 2020. We reported, however, a 49% decrease, yielding to a 34% net decrease in the number of new diagnoses. The strongest impact was estimated in northern regions (−40%) and MSM (−38%). We estimated 761 (95% prediction interval: 350–1277) missed diagnoses during 2020, the majority of them occurring in the North (465 cases), among MSM (416) and heterosexual males (217).

**Conclusions:**

In 2020, when excluding 15% decrease of new diagnoses attributable to the expected reduction, an additional 34% decrease was observed, representing a large decline in new HIV diagnoses associated with the COVID-19 pandemic.

## Introduction

Global COVID-19 pandemic has put a strain on national health systems, having both direct and indirect effects on individual health: disruption of routine medical services or people’s hesitancy in accessing health services, thus causing delays in both primary and secondary prevention.^1^

Among the many consequences of the pandemic in 2020, there was a reduction in the number of HIV diagnoses, as reported in the USA where the number of HIV diagnoses in 2020 was 17% lower than in 2019.^2^ Similarly, in Europe, the count of new diagnoses in 2020 was 24% lower than in 2019.^3^ In Italy, 31% decrease occurred in the number of new HIV diagnoses in 2020 compared to 2019 in Brescia (North Italy, Lombardy region), one of the most COVID-hit Italian provinces in terms of both number of COVID-19 confirmed cases, hospitalized patients and deaths.^4^ The decrease in the number of new HIV diagnoses was mostly attributable to the decrease in HIV testing due to the restriction measures and to the disruption of HIV care services.^5^^,^^6^ In the WHO European Region, a decrease higher than 50% in the number of HIV testing was reported in early 2020 as compared to early 2019.^7^

In Italy, National HIV Surveillance data showed a 56% reduction in the number of new diagnoses in 2020 compared to the number of new diagnoses notified in previous years.^8–11^ However, this reduction did not take account of the historical trend before the pandemic.

The aim of this study was to estimate the expected number of new HIV diagnoses in 2020 based on the previous historical trend, and to compare it with the observed data in 2020 in order to isolate the impact of the COVID-19 pandemic on HIV incidence.

## Methods

### Population and study design

We considered new HIV infections diagnosed from 1 January 2012 to 31 December 2020 reported to the National HIV Surveillance System, updated to December 2021.^8^ Further, data reported before 2012 were not included because the coverage of the National HIV Surveillance System was partial; besides we did not include 2021 diagnoses since that not yet consolidated data, i.e. with reporting delay. In brief, the National HIV Surveillance System collects every year the following information: demographic characteristics (age at diagnosis, gender, nationality, geographical area of diagnosis and geographical area of residence), laboratory and clinical data (first CD4 cell count, first viral load and clinical stage) and transmission mode: people who inject drugs (PWID), heterosexual female, heterosexual male, men who have sex with men (MSM), and other/not available.^10^

### Statistical analysis

We grouped categorical variables by year of HIV diagnosis using frequency and percentages. We calculated percentages with and without missing values in the variables. We estimated population-incidence rates of HIV diagnoses per 100 000 residents.

In order to estimate the expected number of new HIV infections in 2020, we assessed the temporal trend of new HIV diagnoses applying negative binomial mixed effects models to account for over dispersion observed in the distribution of new HIV diagnoses in the period 2012–19.^12^^,^^13^ The yearly count of new HIV diagnoses was the outcome variable in the models, whilst the main covariate was the year of diagnosis from 2012 to 2019, before the COVID-19 pandemic. We entered the region of diagnosis in the models as a random effect (intercept) to consider of the possible clustering at regional level due to differences in health services availability; resident population by region was included as an offset.^14^ In details, i.e. the models were random effects models, with region as random within each macro-area; Northern macro-area includes the following regions: Piemonte, Valle d'Aosta, Lombardia, Tentino-Alto Adige, Veneto, Friuli-Venezia Giulia, Liguria and Emilia-Romagna; the Centre–South–Islands includes: Toscana, Umbria, Marche, Lazio; Abruzzo, Molise, Campania, Puglia, Basilicata, Calabria, Sardegna and Sicilia.

To describe temporal trends of HIV new diagnoses, we used linear splines, i.e. piece-wise linear regression models. For the choice of the knots in the splines, we considered the possible change in the trend due to the introduction of the new guidelines in Italy starting from 2015 (immediate start of treatment regardless of clinical status).^15^^,^^16^ Our choice was supported by published studies showing that immediate start of treatment leads to a lowering of the viral load with consequent decreased probability of HIV transmission, as well as by studies that showed immediate treatment could potentially curb HIV transmission at population level.^17^^,^^18^

Therefore, in the linear splines, we included a knot in 2015 and a second one in 2017, after assessing that the model with two knots showed a better fit to the data, i.e. a lower Akaike’s Information Criterion (AIC) (AIC = 1532), respect to the model with only one knot in 2015 (AIC = 1550). Then, the model used was basically an interrupted time-series analysis with the new ART-treatment guideline as the intervention, and three slopes estimates: the first slope estimating of the time effect before the intervention in 2015 (pre-intervention trend), the second estimating the short-term effect of the intervention effect and a third estimating the longer-term effect of the intervention.^19^

For the multiple analysis, in addition to region of diagnosis, we added the following covariates: age at diagnosis (0–24, 25–49, 50+), HIV-transmission mode (PWID, heterosexual female, heterosexual male, MSM and other/unknown) and CD4 count at diagnosis (<350 cells/mm^3^; ≥350 cells/mm^3^; not reported). In addition, interactions between time and mentioned variables were tested, one by one, and entered in the models when statistically significant (i.e. *P* < 0.05). We applied multiple negative binomial mixed effects models also stratifying by two geographical macro-areas (North and Centre–South–Islands) and by HIV-transmission mode. After graphing the above-described negative binomial mixed effects models, we estimated the expected changes in incidence rates of new HIV diagnoses during recent years with its relative 95% CI, i.e. considering the slope before the pandemic year 2020 estimated using an interruption time-series approach as described above. We first estimated the COVID-19 impact on percentage terms as the difference between the above-mentioned slopes and the change per cent of reported diagnoses by the National HIV Surveillance System (mean change of the two pre-pandemic years). Secondly, we estimated the expected number of new HIV diagnoses in 2020 based on the historical trend estimated, multiplying model-predicted count in year 2019 considering the slope estimated before the pandemic by the negative binomial mixed effects model using an interruption time-series approach. Similarly, we obtained the 95% prediction intervals for the estimates. We finally estimated the number of missed diagnoses during the pandemic year 2020 as the difference between expected and reported diagnoses.

We applied SAS 9.4 for all statistical analyses.

## Results

### Description of the study population

Supplementary table S1 shows the characteristics of the study population consisting of 29 697 new HIV diagnoses reported to the National HIV Surveillance System from 2012 through 2020. New HIV diagnoses decreased from 4176 in 2012 to 1393 in 2020 with an incidence rate of 6.9 and 2.2 per 100 000 residents, respectively. The majority of diagnoses were males (78%) and this proportion remained stable throughout the study period. Most cases were Italian (70%), whilst non-Italians were about 30%.

Adults aged 25–49 were the most represented group although their proportion decreased from 74% in 2012 to 65% in 2020, whereas individuals aged 50+ increased from 17% to 27%, respectively.

Overall, more than 50% of all new HIV diagnoses were reported in northern regions showing a decrease over time. At the opposite, new diagnoses increased in the rest of Italy (Centre–South–Islands), representing about 56% of all diagnoses in 2020.

The highest proportion of diagnoses was represented by MSM (40%), followed by heterosexual males (26%), heterosexual females (19%) and PWID (4%). Furtherly, late diagnoses, i.e. with CD4 < 350 cells/mm^3^, accounted for 56% with an increasing trend that achieved 60% in 2020.

**Figure 1:**
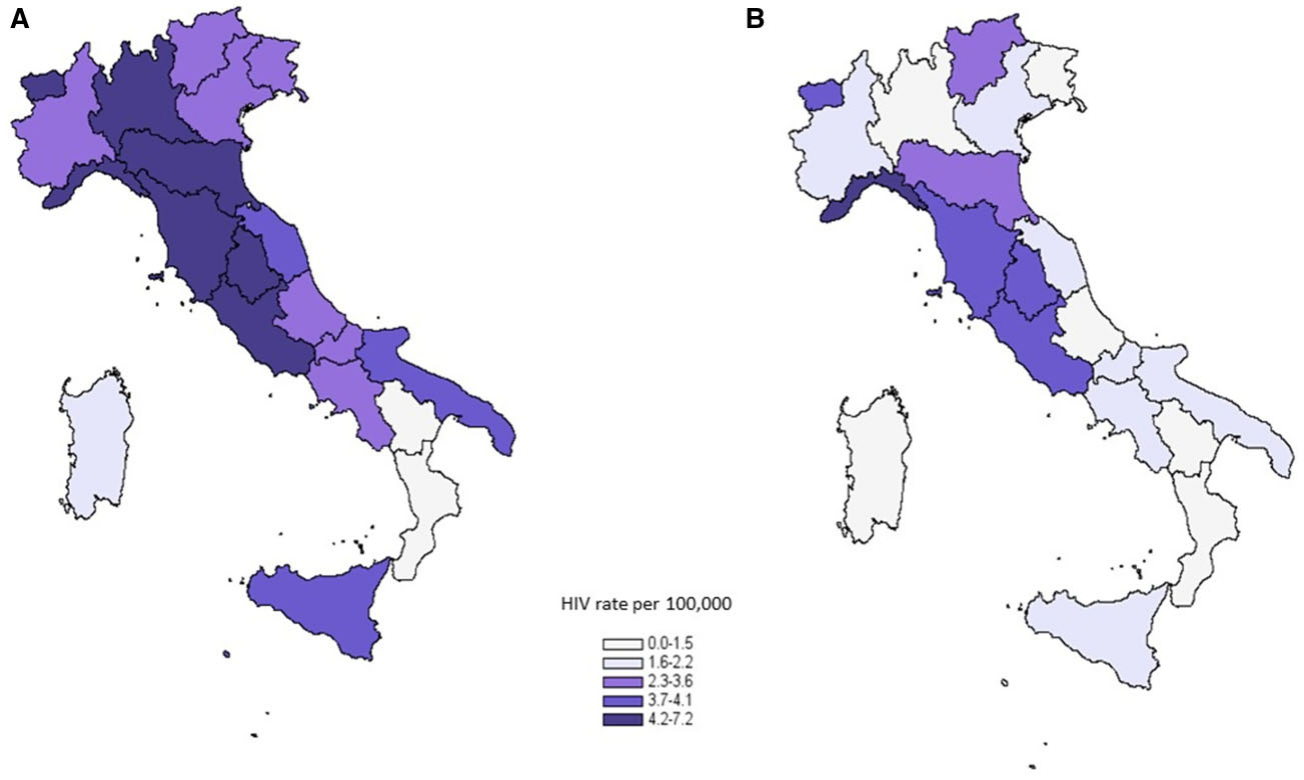
Rates of new HIV diagnoses per 100 000 residents in Italy; (A) Year 2019; (B) Year 2020

When comparing regional incidence rates reported in 2019 (figure 1A) with that in 2020 (figure 1B), we observed a lower rate in 2020 for almost all Italian regions. The decrease in 2020 was more evident in northern regions, such as Lombardy, where the incidence rate decreased from 4.2 in 2019 to 2.3 in 2020 per 100 000 residents.

### Estimate of the historical trend of new HIV diagnoses

**Figure 2:**
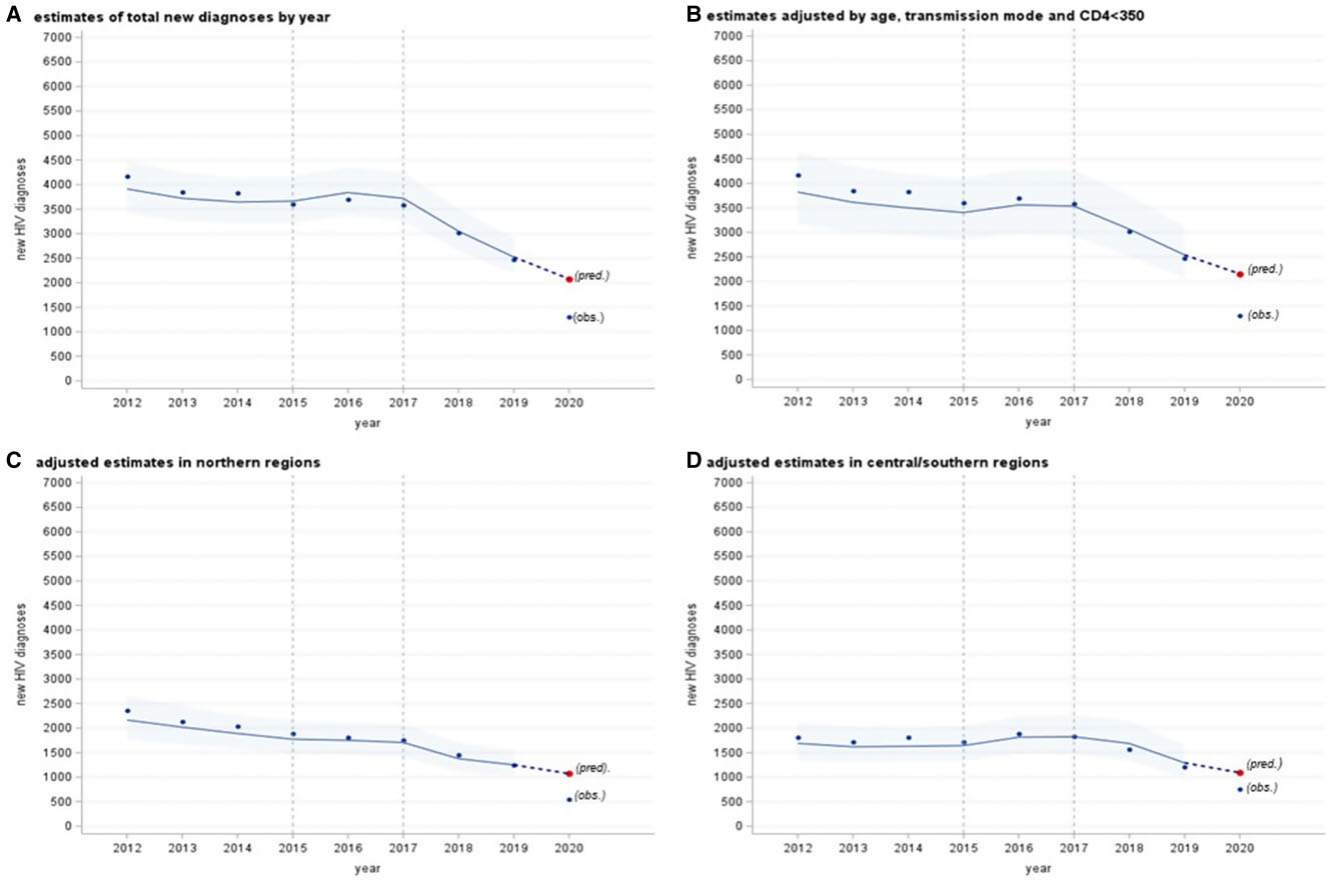
Temporal pattern of new HIV diagnoses and estimates by negative binomial mixed effects models; (A) total diagnoses and estimates with regions as random effect; (B) total diagnoses and estimates by multiple model; (C) total diagnoses in Northern regions and estimates by multiple model; (D) total diagnoses in Centre/Southern regions–Islands and estimates by multiple model; dots, observed (obs.); dot, expected for 2020 (pred.); line, line estimated by models; bands are 95% prediction intervals

Figure 2 shows the temporal pattern of new HIV diagnoses in Italy, as well as the predicted estimates by the model with 95% prediction intervals. Specifically, vertical dotted lines, in 2015 and 2017 show the years where we considered the knots in the splines as described in the statistical analysis. Figure 2A illustrates a decrease in the number of new diagnoses, more pronounced after 2017. The estimated slopes showed a decrement of new HIV diagnoses per year of 3.07% (*P* = 0.084) until 2014, a slight increment of 2.59% (*P* = 0.151) from 2015 to 2016 and a strong reduction thereafter (18.45%, *P* < 0.001). When applying multiple models (figure 2B), the results confirmed a decline in the number of HIV diagnoses mainly since 2017. When stratifying by geographical macro-area, we estimated an almost constant decline in the number of diagnoses after 2012 in northern regions (figure 2C), whilst in the rest of Italy the decrease started after 2017 (figure 2D).

Supplementary figure S2 shows the decreasing incidence trends observed by transmission mode. Among MSM, the decline begun in 2012 and continued afterwards (Supplementary figure S2A). Both heterosexual males and females showed a steady trend from 2012 to 2014, a temporary increase from 2015 to 2016 and a decrease thereafter (Supplementary figure S2B and C). Among PWID, we observed a constant slight decline during the study period, more pronounced before 2015 (Supplementary figure S2D).

### Comparison between expected and reported decrease of new HIV diagnoses in 2020

Table 1 shows the expected decreases (%) of the diagnoses in 2020 based on the historical trend (illustrated in figure 2 and Supplementary figure S2) and reported ones by National HIV Surveillance. Table 1 illustrates also the differences between above decreases, i.e. expected minus reported. Subtracting the 49% reported decrease to the 15% (95% CI: 5–25%) based on the historical trend in 2020, we obtained 34% lower number of diagnoses. As regard main characteristics, estimates show a stronger impact of COVID-19 in the North (−40%), among PWID (−53%), followed by MSM (−38%) and heterosexual males (−31%). Table 2 illustrates, in numerical terms, the comparisons between reported and expected number of new HIV diagnoses in 2020. An expected number of 2154 HIV diagnoses was estimated in 2020, whereas 1393 cases were reported to the Surveillance System. The difference of 761 cases between expected and reported cases was an estimate of the number of missed diagnoses during the pandemic year 2020. The majority of the number of missed diagnoses was estimated in the North (465 cases), among MSM (416), and heterosexual males (217).

**Table 1:** Estimated impact of COVD-19 on new HIV diagnoses in 2020 by main characteristics—Italy, 1 January 2012–31 December 2019; 2021 database

	Expected decrease of the diagnoses in 2020 based on the historical trend (95% CI)^a^	Reported decrease of diagnoses in 2020 respect to the diagnoses registered after 2017 (mean) in the surveillance system (%)	Difference: reported decrease − expected decrease
Total	−15% (−25%; −5%)	−49%	−34%
Geographical area			
North	−15% (−28%; 0%)	−55%	−40%
Centre, South and Islands	−16% (−32%; −2%)	−44%	−28%
Transmission mode			
MSM	−7% (−23%; 0%)	−45%	−38%
Heterosexual male	−17% (−36%; +7%)	−48%	−31%
Heterosexual female	−22% (−41%; 0%)	−52%	−30%
PWID	−1% (−47%; +1%)	−54%	−53%
Not reported	−31% (−65%; 0%)	−65%	−34%

a Expected slopes estimated by multiple negative binomial mixed effects models.

**Table 2:** Estimate of number of missed new HIV diagnoses for the year 2020 by main characteristics—Italy, 1 January 2012–31 December 2020; 2021 database

	No. of HIV diagnoses reported in 2020	Expected no. of HIV diagnoses in 2020 (95% prediction interval)^a^	Estimate of no. of missed HIV diagnoses in 2020 (reported − expected) (95% prediction interval)
Total	1393	2154 (1743; 2670)	−761 (−1277; −350)
Geographical area			
North	607	1072 (856; 1346)	−465 (−739; −249)
Centre–South–Islands	786	1092 (839; 1424)	−306 (−638; −53)
Transmission mode			
MSM	633	1049 (786; 1408)	−416 (−775; −153)
Heterosexual male	351	568 (416; 782)	−217 (−431; −65)
Heterosexual female	241	355 (262; 486)	−114 (−245; −21)
PWID	50	97 (59; 166)	−47 (−116; −9)
Not reported	118	147 (91; 242)	−29 (−124; +27)

a Estimates multiplying model-predicted count in year 2019 by incidence rate ratio (IRR) estimated by the negative binomial mixed effects model.

## Discussion

This study shows in Italy a significant decrease in new HIV diagnoses during the first COVID-19 pandemic year as compared to the previous historical HIV trend. We observed in 2020 a 49% decrease in the number of new HIV diagnoses when comparing to pre-pandemic years. When excluding the 15% decline due to the historical decreasing trend, we estimated a net 34% decrease attributable to the COVID-19 pandemic in 2020.

The estimate of the HIV historical trend, our first objective, was obtained through a negative binomial mixed effects model that used data of the National HIV Surveillance System during pre-COVID-19 period. Before 2020, this model estimated a HIV rate decline of 15% per year (95% CI: −25%; −5%); of note, the decline observed was mainly attributable to: ‘test and treat’ therapeutic strategy (i.e. immediate ART treatment regardless of CD4 count), introduced in Italy in 2015.^15^^,^^16^ This strategy leads in fact to a lowering of the viral load with consequent decreased probability of HIV transmission.^17^^,^^18^ A similar decreasing trend was observed in all countries of EU/EEA area.^11^

Through estimated historical decline before the pandemic, we obtained an estimate of the impact of the COVID-19 on National HIV Surveillance System: in fact, when subtracting expected decline from observed one in 2020, we estimated a net 34% decrease correlated to COVID-19, ranging from a minimum of 24% to a maximum of 44%. In absolute terms, this corresponded to a total missed HIV diagnoses in 2020 of 2154 with a minimum of 1743 and maximum of 2670. To our knowledge, this is the first study in Europe attempting such an estimate and suggesting the net impact of COVID-19 on HIV Surveillance. Only one recent study conducted in USA found a similar effect of the pandemic, however, showing a much lower impact, i.e. 18% fewer diagnoses than expected in 2020.^20^ This can be due to different study populations and methods, but also to the early and strong pandemic that occurred in Italy. Therefore, the estimated impact of COVID-19 pandemic on HIV diagnoses can be associated to a number of factors: first of all, the reduction of HIV testing in 2020, as already reported in Italy; therefore, the majority of WHO European countries reported severe disruptions to testing provision respect to 2019.^4^^,^^5^^,^^7^^,^^21^ Second, the long and strict quarantine measures applied by the Italian Government to contain the spreading of SARS-CoV-2 infection in 2020 may have implied a further decrease of HIV testing due to less risky behaviours or less access to testing services.^22^

We estimated also the decline of HIV diagnoses by main characteristics of the study population, obtaining a similar 15% decline before the pandemic by geographical macro-area. When comparing the reduction observed in 2020 vs. that expected, we estimated a greater decline in the North, (−40% in the North vs. −28% in the rest of Italy). This result confirms the impact of the COVID-19 pandemic that affected mostly northern Italian regions, as published by some studies conducted in the North, known to be the first European hit geographical area.^4^^,^^23^^,^^24^

In terms of HIV-transmission mode, based on the historical trend, we expected the smallest declines in PWID and MSM, −1% and −7%, respectively. Nevertheless, we estimated the highest impact during the pandemic year, specifically −53% in PWID and −38% in MSM. These findings are consistent with other studies that found that lockdowns and social distancing practices reduced HIV testing and syringe services programmes in USA.^25^^,^^26^ As regard MSM, a reduced access to HIV services together with continuation of HIV-risk behaviours was reported by a cohort study conducted among MSM in Italy.^27^ Furthermore, recent studies highlight the COVID-19 impact in decreasing access to the community-based services testing among MSM in the WHO European region, as well as at a population level.^28^^,^^29^ At opposite, due to different historical trends before 2020, we estimated higher recent declines among heterosexuals, with a consequent COVID-19 smaller impact in 2020 (−21% in heterosexual females and −30% in heterosexual males, respectively). On absolute terms, the mentioned declines corresponded to the majority of missed diagnoses in the North (−465), in MSM (−416) and in heterosexual males (−217). The found differences in our estimates between MSM and heterosexuals could reflect the historical decline of diagnoses in MSM that occurred earlier than the other HIV-transmission mode groups, resulting consistent with findings in UK among MSM.^30^^,^^31^ Conversely, heterosexual group showed in this study a more recent decline, i.e. since 2017. Heterosexual males and females showed similar trends in EU/EEA countries; the decline started later among heterosexuals as mainly due to poorer HIV-risk perception compared with MSM during pre-pandemic period; therefore, in Italy, as in other European countries, surveillance data had reported consequently higher proportion of late diagnoses among heterosexuals respect to MSM.^8^^,^^32^^,^^33^

The strength of our study consists in estimating the impact of COVID-19 on new HIV diagnoses, by geographical macro-area and transmission mode, the first in Europe that estimated the impact based on the expected number of HIV diagnoses. The estimates considered the historical trend and were adjusted for important factors, such as age, CD4 at first diagnosis and HIV-transmission model. Moreover, the analysis included all new HIV diagnoses registered to the National HIV Surveillance System, allowing reproducibility of the statistical model to the other EU/EEA countries with similar surveillance data and ensuring comparability with other countries.

Some limitations should be considered, first of all the strong assumption made in the model, i.e. that the historical trend in diagnoses would have continued following the trend in the period 2017–19 in the absence of the pandemic. However, we observed a steady decline of the new diagnoses count after 2020 that is in line with our estimates of the diagnoses before the pandemic.^10^ However, to what extent the post-pandemic trend depends on that before 2020 and/or the effect of the pandemic itself must still be investigated. Another limitation should be the under-reporting and/or reporting delay, which can affect observed data and estimates. However, the impact of reporting delay should be negligible because we used data updated to the end of 2021, in fact in Italy the HIV data are consolidated at 97% 1 year after the diagnosis.^8–10^

Our study shows that, beyond the historical decreasing trend of new HIV diagnoses observed in recent years, the COVID-19 pandemic had a net and strong impact on the HIV epidemic in Italy, greatly affecting northern regions where the pandemic was most serious, and leading to possible several missing diagnoses.

## Supplementary Material

ckad156_Supplementary_Data

## Data Availability

Documentation for National HIV Surveillance System is publicly available at https://www.iss.it/en/-/volume-35-no.-11-november-2022.-hiv/aids-infection-in-italy-up-to-december-31-2021. Data cannot be shared for ethical/privacy reasons. The data underlying this article cannot be shared publicly due to the privacy of the individual cases and GDPR issues. The aggregate anonymized data may be shared on reasonable request to the corresponding author after necessary permissions are sought and obtained. Key points In Western European countries, the COVID-19 impact on HIV surveillance system has not yet been isolated by the historical decline. This study estimated in Italy about 800 missed diagnoses, corresponding to more than twice than expected decline in HIV diagnoses. A strong geographical pattern was found, with 40% fewer HIV diagnoses in northern regions, the most and first European hit geographical area in 2020. More effective measures must be implemented to monitor the HIV incidence, in order to achieve the 95–95–95 UNAIDS targets in all people living with HIV.
