# Supplementary material for: Impact of COVID-19 epidemic on temporal pattern of new HIV diagnoses in Italy, 2021 database
Source: Eur J Public Health. 2023 Aug 31;33(6):1171–6. doi: 10.1093/eurpub/ckad156 (PMC10710354; doi:10.1093/eurpub/ckad156)

| **Supplementary Table S1 - Characteristics of new HIV diagnoses by year - Italy, Jan 1st 2012 - Dec 31st 2020; 2021 database.** | | | | | | | | | | | | | | | | | | | | | |
| --- | --- | --- | --- | --- | --- | --- | --- | --- | --- | --- | --- | --- | --- | --- | --- | --- | --- | --- | --- | --- | --- |
|  |  | **2012** | | **2013** | | **2014** | | **2015** | | **2016** | | **2017** | | **2018** | | **2019** | | **2020** | | ***Total*** | |
|  |  | **n.** | **%^a^** | **n.** | **%^a^** | **n.** | **%^a^** | **n.** | **%^a^** | **n.** | **%^a^** | **n.** | **%^a^** | **n.** | **%^a^** | **n.** | **%^a^** | **n.** | **%^a^** | **n.** | **%^a^** |
| **Gender** |  |  |  |  |  |  |  |  |  |  |  |  |  |  |  |  |  |  |  |  |  |
| Male |  | 3,288 | *78.7* | 3,006 | *78.0* | 3,052 | *79.5* | 2,800 | *77.5* | 2,854 | *76.9* | 2,736 | *76.0* | 2,361 | *78.3* | 1,990 | *79.9* | 1,111 | *79.8* | *23,198* | *78.1* |
| Female |  | 888 | *21.3* | 847 | *22.0* | 789 | *20.5* | 815 | *22.5* | 857 | *23.1* | 863 | *24.0* | 656 | *21.7* | 502 | *20.1* | 282 | *20.2* | *6,499* | *21.9* |
| **Nazionality** | |  |  |  |  |  |  |  |  |  |  |  |  |  |  |  |  |  |  |  |  |
| Italian |  | 3,015 | *72.6* | 2,889 | *75.1* | 2,758 | *72.1* | 2,561 | *71.2* | 2,381 | *64.4* | 2,331 | *65.0* | 2,108 | *70.2* | 1,807 | *72.7* | 938 | *67.7* | *20,788* | *70.3* |
| Non Italian | | 1,139 | *27.4* | 956 | *24.9* | 1,069 | *27.9* | 1,038 | *28.8* | 1,317 | *35.6* | 1,256 | *35.0* | 894 | *29.8* | 680 | *27.3* | 447 | *32.3* | *8,796* | *29.7* |
| Not reported^b^ | | 22 | *0.5* | 8 | *0.2* | 14 | *0.4* | 16 | *0.4* | 13 | *0.4* | 12 | *0.3* | 15 | *0.5* | 5 | *0.2* | 8 | *0.6* | *113* | *0.4* |
| **Age group** | |  |  |  |  |  |  |  |  |  |  |  |  |  |  |  |  |  |  |  |  |
| <25 |  | 356 | *8.5* | 310 | *8.0* | 363 | *9.5* | 316 | *8.7* | 408 | *11.0* | 460 | *12.8* | 297 | *9.8* | 183 | *7.4* | 107 | *7.7* | *2,800* | *9.4* |
| 25-49 |  | 3,099 | *74.2* | 2,819 | *73.2* | 2,740 | *71.3* | 2,575 | *71.2* | 2,524 | *68.0* | 2,329 | *64.7* | 1,974 | *65.5* | 1,621 | *65.2* | 905 | *65.0* | *20,586* | *69.3* |
| 50+ |  | 720 | *17.2* | 724 | *18.8* | 738 | *19.2* | 724 | *20.0* | 778 | *21.0* | 810 | *22.5* | 745 | *24.7* | 683 | *27.5* | 381 | *27.4* | *6,303* | *21.2* |
| Not reported^b^ | | 1 | *0.0* | 0 | *0.0* | 0 | *0.0* | 0 | *0.0* | 1 | *0.0* | 0 | *0.0* | 1 | *0.0* | 5 | *0.2* | 0 | *0.0* | *8* | *0.0* |
| **Geographical area** | |  |  |  |  |  |  |  |  |  |  |  |  |  |  |  |  |  |  |  |  |
| North |  | 2,366 | *56.7* | 2,129 | *55.3* | 2,035 | *53.0* | 1,894 | *52.4* | 1,819 | *49.0* | 1,761 | *48.9* | 1,448 | *48.0* | 1,262 | *50.6* | 607 | *43.6* | *15,321* | *51.6* |
| Centre |  | 1,090 | *26.1* | 1,058 | *27.5* | 1,098 | *28.6* | 970 | *26.8* | 1,101 | *29.7* | 950 | *26.4* | 797 | *26.4* | 624 | *25.0* | 442 | *31.7* | *8,130* | *27.4* |
| South and Islands | | 720 | *17.2* | 666 | *17.3* | 708 | *18.4* | 751 | *20.8* | 791 | *21.3* | 888 | *24.7* | 772 | *25.6* | 606 | *24.3* | 344 | *24.7* | *6,246* | *21.0* |
| **Transmission mode** | |  |  |  |  |  |  |  |  |  |  |  |  |  |  |  |  |  |  |  |  |
| MSM |  | 1,601 | *38.3* | 1,522 | *39.5* | 1,566 | *40.8* | 1,460 | *40.4* | 1,414 | *38.1* | 1,381 | *38.4* | 1,180 | *39.1* | 1,106 | *44.4* | 633 | *45.4* | *11,863* | *39.9* |
| Heterosexual male | | 1,058 | *25.3* | 992 | *25.7* | 1,006 | *26.2* | 941 | *26.0* | 1,023 | *27.6* | 904 | *25.1* | 704 | *23.3* | 641 | *25.7* | 351 | *25.2* | *7,620* | *25.7* |
| Heterosexual female | | 711 | *17.0* | 714 | *18.5* | 659 | *17.2* | 702 | *19.4* | 740 | *19.9* | 749 | *20.8* | 545 | *18.1* | 450 | *18.1* | 241 | *17.3* | *5,511* | *18.6* |
| PWID |  | 219 | *5.2* | 182 | *4.7* | 142 | *3.7* | 121 | *3.3* | 112 | *3.0* | 103 | *2.9* | 113 | *3.7* | 103 | *4.1* | 50 | *3.6* | *1,145* | *3.9* |
| Other/Unknown | | 587 | *14.1* | 443 | *11.5* | 468 | *12.2* | 391 | *10.8* | 422 | *11.4* | 462 | *12.8* | 475 | *15.7* | 192 | *7.7* | 118 | *8.5* | *3,558* | *12.0* |
| **CD4 cell count** | |  |  |  |  |  |  |  |  |  |  |  |  |  |  |  |  |  |  |  |  |
| <350 cell/μL | | 1,796 | *54.8* | 1,703 | *56.7* | 1,600 | *53.4* | 1,545 | *54.4* | 1,587 | *54.9* | 1,578 | *55.6* | 1,396 | *56.6* | 1,351 | *58.3* | 788 | *60.2* | *13,344* | *55.7* |
| ≥350 cell/μL | | 1,484 | *45.2* | 1,299 | *43.3* | 1,395 | *46.6* | 1,297 | *45.6* | 1,305 | *45.1* | 1,262 | *44.4* | 1,071 | *43.4* | 967 | *41.7* | 522 | *39.8* | *10,602* | *44.3* |
| Not reported^b^ | | 896 | *21.5* | 851 | *22.1* | 846 | *22.0* | 773 | *21.4* | 819 | *22.1* | 759 | *21.1* | 550 | *18.2* | 174 | *7.0* | 83 | *6.0* | *5,751* | *19.4* |
| **AIDS diagnosis** | |  |  |  |  |  |  |  |  |  |  |  |  |  |  |  |  |  |  |  |  |
| No |  | 2,352 | *78.6* | 2,086 | *77.8* | 2,169 | *78.6* | 2,109 | *78.7* | 2,157 | *77.9* | 2,137 | *79.1* | 1,747 | *77.7* | 1,692 | *76.0* | 907 | *73.7* | *17,356* | *77.9* |
| Yes |  | 640 | *21.4* | 596 | *22.2* | 589 | *21.4* | 571 | *21.3* | 612 | *22.1* | 565 | *20.9* | 502 | *22.3* | 533 | *24.0* | 324 | *26.3* | *4,932* | *22.1* |
| Not reported^b^ | | 1,184 | *28.4* | 1,171 | *30.4* | 1,083 | *28.2* | 935 | *25.9* | 942 | *25.4* | 897 | *24.9* | 768 | *25.5* | 267 | *10.7* | 162 | *11.6* | *7,409* | *24.9* |
| ***Total*** |  | ***4,176*** | ***100*** | ***3,853*** | ***100*** | ***3,841*** | ***100*** | ***3,615*** | ***100*** | ***3,711*** | ***100*** | ***3,599*** | ***100*** | ***3,017*** | ***100*** | ***2,492*** | ***100*** | ***1,393*** | ***100*** | ***29,697*** | ***100*** |
| a: proportion calculated on complete data excluding missing values  b: proportion of missing values calculated on total of diagnoses | | | | | | | | | | | | | | | | | | | | | |
|  |  |  |  |  |  |  |  |  |  |  |  |  |  |  |  |  |  |  |  |  |  |

**Supplementary Figure S2.** Temporal pattern of new HIV diagnoses and estimates by negative binomial mixed effects models; Italy, 2012-2020; part A) diagnoses in MSM; part B) diagnoses in heterosexual males; part C) diagnoses in heterosexual females; part D) diagnoses in PWID; blue dots = observed cases (obs.); red dot = expected cases in 2020 (expect.); line = trend estimated by models; bands are 95% prediction intervals


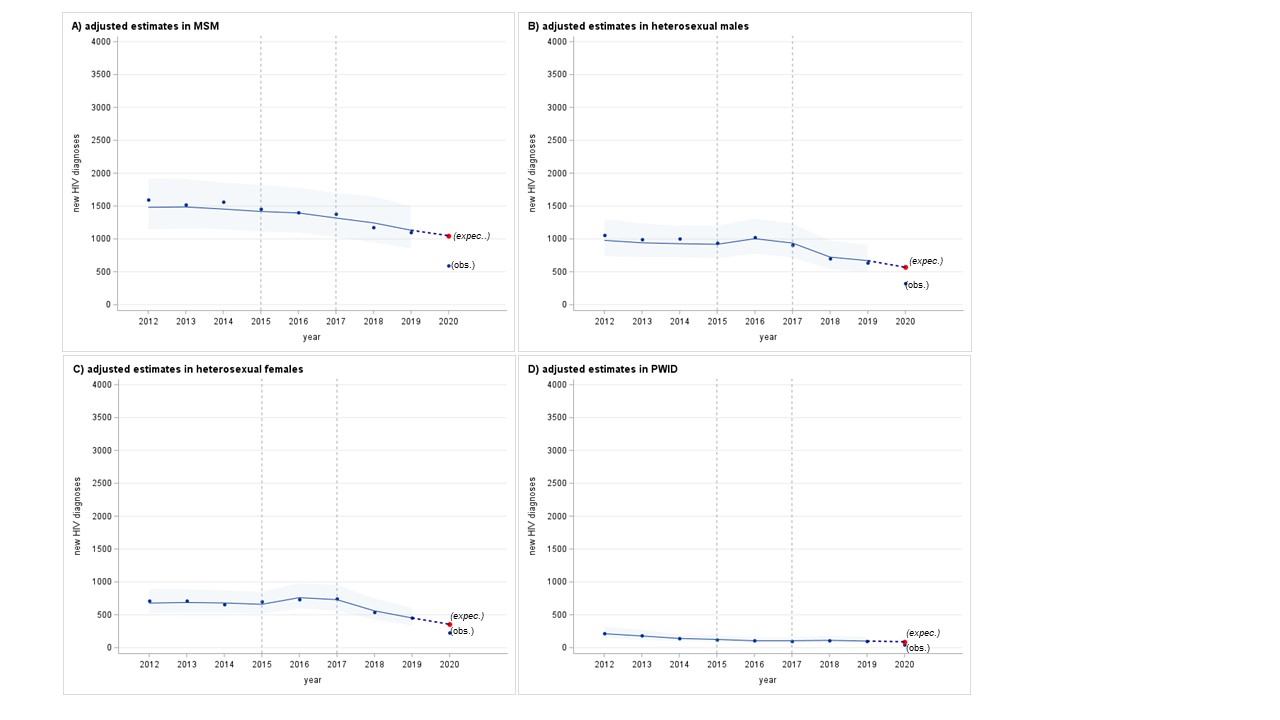

Supplement: ckad156_Supplementary_Data [file ckad156_supplementary_data.docx]
